# The Association of Nocturnal Blood Pressure Patterns and Other Influencing Factors With Lacunes and Enlarged Perivascular Spaces in Hypertensive Patients

**DOI:** 10.3389/fneur.2022.879764

**Published:** 2022-05-23

**Authors:** Yang Gao, Weiping Deng, Jialan Sun, Dongqi Yue, Bei Zhang, Yulan Feng, Jun Han, Fanxia Shen, Jin Hu, Yi Fu

**Affiliations:** ^1^Department of Neurology and Institute of Neurology, Ruijin Hospital, Shanghai Jiao Tong University School of Medicine, Shanghai, China; ^2^Department of Neurology, The First Hospital of Jiaxing and The Affiliated Hospital of Jiaxing University, Jiaxing, China; ^3^Department of Neurology, Minhang Hospital, Fudan University, Shanghai, China; ^4^Department of Radiology, Ruijin Hospital, School of Medicine, Shanghai Jiao Tong University, Shanghai, China; ^5^Department of Radiology, The First Hospital of Jiaxing and The Affiliated Hospital of Jiaxing University, Jiaxing, China; ^6^Department of Neurology, Pudong New Area Gongli Hospital, Shanghai, China

**Keywords:** lacunes, ePVS, enlarged perivascular spaces, nocturnal blood pressure decline, dipper, non-dipper, extreme-dipper, reverse-dipper

## Abstract

**Purpose:**

Nocturnal blood pressure dipping patterns have been associated with an increased risk of Cerebral Small Vessel Disease (CSVD), which has not been well-studied. This study is aimed to explore the association of dipping patterns and other factors with lacunes and enlarged perivascular spaces (EPVS) in patients with hypertension.

**Methods:**

We enrolled a total of 1,322 patients with essential hypertension in this study. Magnetic resonance imaging (MRI) scans and 24-h ambulatory blood pressure (BP) monitoring were completed. Nocturnal BP decline was calculated, and then dipping patterns were classified. Patients were classified into four groups according to the performance of lacunes and EPVS in the MRI scan for statistical analysis.

**Results:**

(1) Nocturnal BP decline showed independent negative correlation with both lacunes and EPVS while mean systolic BP (mSBP) level showed an independent positive correlation with them (*P* < 0.05). (2) The frequency of reverse-dippers in the control group was significantly lower than that in other groups; the frequency of non-dippers in the lacunes group and EPVS group was significantly lower than that in the control group; the frequency of extreme-dippers in the EPVS group was significantly higher than that in the mixed (lacunes with EPVS) group (*P* < 0.05).

**Conclusions:**

Both mSBP and dipping patterns might play an important role in developing lacunes and EPVS in patients with hypertension.

## Introduction

Cerebral Small Vessel Disease (CSVD) is a type of disease that affects small vessels in the brain (1), manifesting as lacunes, white matter hyperintensities (WMHs), enlarged perivascular spaces (EPVS), and cerebral microbleeds (CMBs) (2). More prevalent in the elderly, CSVD has been shown to account for about 25% of ischemic stroke (1). By now, neuroimaging examinations, especially magnetic resonance imaging (MRI), are widely applied for the discovery and differentiation of CSVD patients. However, the silent nature of CSVD at the very early state hampered the full understanding of its pathogenesis of CSVD and the specific prevention and treatment methods (1).

Lacunes are small fluid-filled cavities (between 3 and 15 mm in diameter) that are thought to mark the healed stage of a small deep brain infarct (1). Perivascular spaces (PVS), also known as the Virchow-Robin spaces, are normally referred to as the potential spaces that surround the arterioles, capillaries, and venules. EPVS are referred to as MRI-visible PVS (3).

The blood pressure (BP) fluctuates throughout the day, usually higher during the daytime and lower during the nighttime. Physiological fluctuations of BP are important for health maintenance (4). The disordered circadian rhythm of BP is considered to be a significant risk factor for cerebrovascular diseases (5).

Previous studies have focused on the relationship between hypertension and cerebral small vessel disease, yet the relationship between nocturnal dipping patterns and EPVS has rarely been reported. It was reported that nocturnal non-dipping of blood pressure and heart rate were associated with an increased risk of silent stroke and CSVD (6), while higher SBP levels were independently associated with EPVS in Basal ganglia, but not in centrum semiovale (7). Though patients with lacunes or EPVS could have few clinical manifestations, they may have an increased risk for multiple serious diseases in the future (5). It was reported that patients with lacunes were more likely to develop dementia (8), while patients with EPVS were more susceptible to developing lacunar infarction (9), vascular dementia (10), and recurrent lobar hemorrhage (11). Additionally, the total small vessel disease burden score was higher in patients with both EPVS and lacunes, indicating a higher hazard of future stroke, and a worse functional prognosis (12). To our knowledge, hypertension is the most important risk factor for CSVD, and both lacunes and EPVS are common in patients with hypertension. Therefore, in this study, we aimed to explore the association of dipping patterns with lacunes and EPVS in hypertensive patients.

## Methods

### Study Population and Ethical Approval

We enrolled hypertensive patients who were admitted to the Department of Neurology, Department of Cardiology or Department of Hypertension of Ruijin Hospital Affiliated to Shanghai Jiao Tong University School of Medicine, Minhang Hospital Affiliated to Fudan University, and The First Hospital of Jiaxing from February 2015 to February 2019. A total of 1,322 hypertensive patients were included in our study.

The inclusion criteria were as follows: older than 18 (included) years with no gender limitation; diagnosed with essential hypertension for at least 1 year; taking antihypertensive drugs regularly with relatively stable BP; relatively complete medical histories with negative physical examination of the nervous system. The exclusion criteria were as follows: definite past histories of stroke; MRI scanning contraindications; secondary hypertension; acute neurology/cardiology event such as myocardial infarction in hospital.

All data of this study was from our database, which was approved by the Medical Ethics Committee of Ruijin Hospital. All methods were performed in accordance with relevant guidelines and regulations. Informed consent was obtained from all subjects or their legal guardians.

### Clinical Data Collection

The clinical information of enrolled hypertensive patients was recorded and confirmed by the attending physicians, which included admission number, admission date, gender, and age. Past medical histories included hypertension duration, family history of hypertension, smoking and drinking history, diabetes mellitus (any type), atrial fibrillation, and long-term administration of medications (such as antiplatelet, anticoagulants, statins). Patients would take their antihypertensive and other regular medications as usual during their hospitalization.

A carotid artery ultrasound was performed to assess whether there were any carotid artery plaques, and an electrocardiograph (ECG) was performed as a supplement to the history of atrial fibrillation. In the morning after admission, fasting blood tests of cholesterol, triglyceride, high-density lipoprotein cholesterol (HDL-c), low-density lipoprotein cholesterol (LDL-c), blood glucose, urea nitrogen, creatinine, homocysteine, and C-reactive protein (CRP) were examined.

The 24-h non-invasive ambulatory blood pressure (ABP) monitoring was completed for patients during their hospitalization by ABP-monitor (type 90217, Spacelabs, USA, Oscillometry). The sphygmomanometer cuff was worn on the upper left arms of the patients. The readings were performed every 30 min during the daytime (06:00–22:00), and every 60 min during the nighttime (22:00–06:00). Nurses regularly patrolled the ward to ensure that patients were wake and asleep at the above time as much as possible. Indexed including mean systolic blood pressure (mSBP), mean diastolic blood pressure (mDBP), mean daytime artery pressure (mDAP), and mean nighttime artery pressure (mNAP) were recorded. Nocturnal BP decline was calculated as follows: (mDAP – mNAP) / mDAP. The dipping patterns were defined by Nocturnal BP decline value: <0 was the reverse-dipper; 0–0.1 was the non-dipper; 0.1–0.2 was the dipper; and >0.2 was the extreme-dipper. The reverse-dipper, non-dipper, and extreme-dipper were defined as the abnormal dipping patterns.

### MRI Analysis

During the hospitalization, axial images of various sequences including T1-weighted, T2-weighted, T2-fluid-attenuated inversion recovery (T2-FLAIR), diffusion-weighted imaging (DWI), and susceptibility-weighted imaging (SWI) were collected using the GE Signa HDxT 3.0 T Superconducting MRI system. Two senior radiologists with significant experience were responsible for the imaging reading work to determine whether there were lacunes and EPVS on the MRI scan. In addition, they needed to count the number and assess EPVS severity. If the results were inconsistent, these two radiologists would discuss them with each other and come to a unified conclusion.

The image interpretation criteria for lacunes were as follows: wedged, round, or ovoid cavities; 3–15 mm in diameter; low T1-weighted signal and high T2-weighted signal in the center, with a hyperintensity rim on T2-FLAIR sequence (1). The image interpretation criteria for EPVS were as follows: circular, ovoid, linear, or tubular structures with clear boundaries on the MRI imaging; consistent with the route of the perforating vessels; cerebrospinal fluid-like signal; without mass effects; less than 3 mm in diameter. The section with the largest number of EPVS in basal ganglia and centrum semiovale was selected to count the single-layer number of EPVS. The existence of EPVS was defined as at least one EPVS in any region. According to the number, the EPVS severity of these 2 regions was classified separately as follows: level 0, no EPVS; level 1, 1–10 EPVS; level 2, 11–20 EPVS; level 3, 21–40 EPVS; and level 4, >40 EPVS (13).

Based on the presence or absence of lacunes and EPVS, all patients were divided into four groups: the lacunes group (patients with only lacunes); EPVS group (patients with only EPVS); mixed group (patients with both lacunes and EPVS); and the control group. Among the EPVS group, patients were further classified according to the severity of EPVS in different regions: EPVS in centrum semiovale predominance (EPVS in centrum semiovale score > EPVS in basal ganglia score); EPVS in basal ganglia predominance (EPVS in basal ganglia score > EPVS in centrum semiovale score); and non-predominance (EPVS in basal ganglia score = EPVS in centrum semiovale score) (14).

### Statistics

SPSS 26.0 software was used for statistical analysis. Continuous variables were tested by Kolmogorov–Smirnov normality analysis. For continuous variables following the normal distribution, variables were represented as $\bar{\text{x}}\pm$ s, and single-factor analysis of variance (ANOVA) was adopted for the comparison among groups. For continuous variables that did not fit the normal distribution, variables were represented as median (interquartile range), and the Kruskal–Wallis *H*-test was adopted for the comparison. Enumeration data were represented by the form of frequency and percentage, and comparisons among groups were analyzed by either χ^2^-test or Fisher's exact test. In the above comparison, we set 0.05 as the significance level. For relevant influencing factors with intergroup differences found above, the univariate Logistic regression was performed separately in the control group and the lacunes/EPVS/mixed group [to calculate the odds ratio (OR) of each factor]. Factors whose *P*-values were less than 0.05 in the univariate logistic regression tests, would be further taken together to find out the independent influencing factors by multivariate logistic regression (Probability for stepwise: entry 0.05, removal 0.10. Method: backward conditional). Among the three predominance types in the EPVS group, the independent influencing factors of EPVS were further compared. A bar chart was drawn according to the dipping patterns in different groups, and the differences were compared by either Pearson's chi test or Fisher's exact test (Bonferroni correction was used for pairwise comparison).

## Results

A total of 1,322 hypertensive patients who met the criteria were included in this study. There were 317 patients in the control group (age, 57 ± 11; males, 42.6%), 358 patients in the lacunes group (age, 66 ± 13; males, 52.2%), 395 patients in the EPVS group (age, 69 ± 12; males, 48.1%), and 252 patients in the mixed group (age, 71 ± 12; males, 57.9%).

### Baseline Data Comparison

Factors including age, hypertension duration, mSBP, mDBP, HDL-c, LDL-c, and fasting blood glucose (FBG) followed the normal distribution. The factors that did not conform to normal distribution were the Nocturnal BP decline, triglyceride, urea nitrogen, creatinine, homocysteine, and CRP.

Among the four groups, factors that showed statistically significant differences *(P* < 0.05) were age, gender, smoking, diabetes mellitus, antiplatelet, statins, cholesterol, hypertension duration, the Nocturnal BP decline, mSBP, triglycerides, HDL-c, LDL-c, urea nitrogen, creatinine, homocysteine, and carotid artery plaque. However, there were no statistical difference in other factors including drinking, atrial fibrillation, anticoagulants, family history of hypertension, mDBP, FBG, and CRP between groups (*P* > 0.05; Table 1).

**Table 1:** The comparison of demographic and clinical characteristics among groups.

**Variables**	**Control (*n* = 317)**	**Lacunes (*n* = 358)**	**EPVS (*n* = 395)**	**Mixed (*n* = 252)**	***P-*value**
**Demographic data and medical history**	
Age (years, $\bar{\text{x}}$ ± s)	57 ± 11	66 ± 13	69 ± 12	71 ± 12	<0.001
Male (*n*, %)	135 (42.6)	187 (52.2)	190 (48.1)	146 (57.9)	0.002
Smoking (*n*, %)	48 (15.1)	84 (23.5)	75 (19.0)	65 (25.8)	0.006
Drinking (*n*, %)	33 (10.4)	62 (17.4)	52 (13.2)	40 (15.9)	0.055
Diabetes mellitus (*n*, %)	46 (14.5)	120 (33.5)	143 (36.2)	91 (36.1)	<0.001
Atrial fibrillation (*n*, %)	5 (1.6)	10 (2.8)	16 (4.1)	14 (5.6)	0.053
Antiplatelet (*n*, %)	55 (17.4)	21 (6.2)	24 (6.1)	15 (6.4)	<0.001
Anticoagulants (*n*, %)	2 (0.6)	2 (0.6)	9 (2.3)	4 (1.7)	0.118
Statins (*n*, %)	63 (19.9)	48 (13.4)	36 (9.1)	36 (14.3)	0.001
**Hypertension associated factors**	
Family history (*n*, %)	93 (29.3)	97 (27.1)	93 (23.5)	57 (22.6)	0.189
Duration (years, $\bar{\text{x}}$ ± s)	7.5 ± 7.3	12.9 ± 10.9	13.8 ± 10.5	14.6 ± 11.4	<0.001
Nocturnal blood pressure decline (%, M, IQR)	8.4 (1.2–15.7)	5.7 (−7.0 ~ 15.6)	5.2 (−6.1 ~15.3)	2.9 (−9.8 ~ 12.9)	<0.001
Mean systolic blood pressure (mmHg, $\bar{\text{x}}$ ± s)	127.6 ± 14.7	132.1 ± 16.7	135.8 ± 17.6	135.9 ± 18.3	<0.001
Mean diastolic blood pressure (mmHg, $\bar{\text{x}}$ ± s)	76.2 ± 10.2	74.1 ± 10.8	74.9 ± 10.7	74.6 ± 11.3	0.082
**Medical examinations**
Cholesterol (mmol/L, $\bar{\text{x}}$ ± s)	4.6 ± 1.1	4.4 ± 1.1	4.4 ± 1.2	4.2 ± 1.4	0.007
Triglycerides (mmol/L, M, IQR)	1.4 (1.0 ~ 1.9)	1.6 (1.2 ~ 2.3)	1.5 (1.1–2.2)	1.5 (1.0~2.4)	0.007
High-density lipoprotein cholesterol (mmol/L, $\bar{\text{x}}$ ± s)	1.3 ± 0.8	1.2 ± 0.4	1.1 ± 0.3	1.1 ± 0.4	<0.001
Low density lipoprotein cholesterol (mmol/L, $\bar{\text{x}}$ ± s)	2.9 ± 0.9	2.7 ± 0.9	2.8 ± 0.9	2.6 ± 1.0	<0.001
Fasting blood glucose (mmol/L, $\bar{\text{x}}$ ± s)	5.7 ± 2.0	5.8 ± 1.8	5.9 ± 2.0	6.0 ± 2.8	0.221
Urea nitrogen (mmol/L, M, IQR)	5.1 (4.2 ~ 6.5)	5.2 (4.4 ~ 6.2)	5.5 (4.9 ~ 6.9)	5.5 (4.4~6.9)	0.013
Creatinine (mmol/L, M, IQR)	62.0 (51.0 ~ 77.8)	73.0 (59.0 ~ 84.0)	72.0 (59.0 ~ 88.0)	77.5 (65.0 ~ 96.3)	<0.001
Homocysteine (μmol/L, M, IQR)	10.5 (8.8 ~ 13.9)	11.8 (10.1 ~ 14.8)	12.2 (10.0 ~ 15.6)	14.1 (11.0 ~ 18.2)	<0.001
C-reactive protein (μg/ml, M, IQR)	1.4 (0.6 ~ 3.8)	2.0 (0.7 ~ 4.0)	1.9 (0.7 ~ 4.4)	1.5 (0.5 ~ 3.9)	0.510
Carotid artery plaque (*n*, %)	74 (23.4)	193 (53.9)	262 (66.3)	180 (71.6)	<0.001

*EPVS, enlargement perivascular spaces, variables were presented as the form of average ± standard deviation, median (interquartile range) or frequency (percentage), and statistically analyzed by ANOVA, Kruskal–Wallis H-test, Pearson Chi square test or Fisher's exact test*.

### The Analysis of Univariate Logistic Regression

The univariate logistic regression was tested in each group separately. The results showed that: (1) Age, male, smoking, diabetes mellitus, mSBP, creatinine, homocysteine, carotid artery plaques, antiplatelet, statins, the Nocturnal BP decline, cholesterol, HDL-c, and urea nitrogen were the potential independent influencing factors for lacunes (*P* < 0.05). (2) Age, smoking, diabetes mellitus, mSBP, creatinine, homocysteine, carotid artery plaque, antiplatelet, statins, the Nocturnal BP decline, HDL-c, and urea nitrogen were the potential independent influencing factors for EPVS (*P* < 0.05). (3) Age, being male, smoking, diabetes mellitus, mSBP, homocysteine, carotid artery plaque, antiplatelet, the Nocturnal BP decline, and cholesterol were the potential independent influencing factors for lacunes and EPVS coexistence (*P* < 0.05; Table 2).

**Table 2:** Univariate logistic regression analysis between groups.

	**Lacunes vs. control**		**EPVS vs. control**		**Mixed vs. control**	
	**OR**	***P*-value**	**OR**	***P*-value**	**OR**	***P*-value**
Age	1.061	<0.001	1.051	<0.001	1.046	<0.001
Male	1.474	0.012	1.250	0.142	1.588	0.002
Smoking	1.551	0.001	1.090	<0.001	1.693	0.003
Diabetes mellitus	2.970	<0.001	3.343	<0.001	1.579	0.004
Antiplatelet	0.310	<0.001	0.316	<0.001	0.554	0.043
Statins	0.624	0.024	0.404	<0.001	0.998	0.992
Nocturnal blood pressure decline	0.111	<0.001	0.109	<0.001	0.102	<0.001
Mean systolic blood pressure	1.014	0.004	1.028	<0.001	1.011	0.010
Cholesterol	0.848	0.023	0.880	0.057	0.898	0.024
Triglycerides	1.113	0.057	1.067	0.205	1.002	0.965
High-density lipoprotein cholesterol	0.666	0.019	0.437	<0.001	0.706	0.085
Urea nitrogen	0.950	<0.001	0.967	<0.001	0.979	0.063
Creatinine	1.011	<0.001	1.013	<0.001	1.001	0.288
Homocysteine	1.056	0.030	1.075	0.006	1.020	0.001
Carotid artery plaque	3.837	<0.001	6.453	<0.001	2.584	<0.001

*EPVS, enlargement perivascular spaces. For each factor, OR value and P-value were presented for each comparison (Lacunes vs. control, EPVS vs. control and Mixed vs. control). Factors whose P-values were less than 0.05 would be further taken together to multivariate logistic regression*.

### The Analysis of Multivariate Logistic Regression

In the multivariate logistic regression models, it was found that mSBP, age, smoking, and diabetes mellitus were the independent positive correlation factors for lacunes, while the Nocturnal BP decline, antiplatelet, and statins showed independent negative correlation with lacunes (*P* < 0.05; Table 3).

**Table 3:** The multivariate logistic regression analysis between lacunes and control.

	**OR**	**95% CI**	***P*-value**
Nocturnal blood pressure decline	0.155	0.075 ~ 0.321	<0.001
Mean systolic blood pressure	1.014	1.008 ~ 1.020	0.021
Age	1.070	1.061 ~ 1.080	<0.001
Antiplatelet	0.228	0.161 ~ 0.324	<0.001
Statins	0.571	0.427 ~ 0.763	0.053
Smoking	3.561	2.773 ~ 4.572	<0.001
Diabetes mellitus	2.385	1.902 ~ 2.989	<0.001

*For each factor, OR value, 95%CI of OR, P-value were presented. The factors whose P-values were higher than 0.05 in univariate logistic regression analysis (Table 2) were excluded in this table*.

For EPVS, the independent positive correlation factors were mSBP and age, and the independent negative correlation factors were the Nocturnal BP decline, antiplatelet, and statins (*P* < 0.05; Table 4).

**Table 4:** The multivariate logistic regression analysis between EPVS and control.

	**OR**	**95% CI**	***P*-value**
Nocturnal blood pressure decline	0.098	0.034 ~ 0.287	0.030
Mean systolic blood pressure	1.024	1.010 ~ 1.034	0.016
Age	1.070	1.059 ~ 1.082	<0.001
Antiplatelet	0.297	0.183 ~ 0.482	0.012
Statins	0.261	0.173 ~ 0.394	0.001

*EPVS, enlargement perivascular spaces. For each factor, OR value, 95%CI of OR, P-value were presented. The factors whose P-values were higher than 0.05 in univariate logistic regression analysis (Table 2) were excluded in this table*.

For lacunes combined with EPVS, factors including mSBP, age, smoking, and homocysteine were independent positive correlation factors. The Nocturnal BP decline was, however, the only independent negative correlation factor for these two lesions (*P* < 0.05; Table 5).

**Table 5:** The multivariate logistic regression analysis between mixed and control.

	**OR**	**95% CI**	***P*-value**
Nocturnal blood pressure decline	0.214	0.113 ~ 0.406	0.016
Mean systolic blood pressure	1.011	1.005 ~ 1.017	0.052
Age	1.027	1.019 ~ 1.035	0.001
Smoking	1.765	1.423 ~ 2.188	0.008
Homocysteine	1.017	1.010 ~ 1.022	0.006

*For each factor, OR value, 95%CI of OR, P-value were presented. The factors whose P-values were higher than 0.05 in univariate logistic regression analysis (Table 2) were excluded in this table*.

### Comparison of Independent Factors Among Three Predominance Types of EPVS

Among the EPVS group, the independent factors discovered by multivariate logistic regression models were further analyzed. The results showed that there were no significant differences of Nocturnal BP decline, mSBP, antiplatelet, statins, and age in patients among three predominance types of EPVS (*P* < 0.05; Table 6).

**Table 6:** The comparison of independent factors among predominance types of EPVS.

	**EPVS in basal ganglia**	**EPVS in centrum semiovale**	**Non-predominance**	***P-*value**
	**predominance (*n* = 99)**	**predominance (*n* = 67)**	**(*n* = 229)**	
Nocturnal blood pressure decline (%, M, IQR)	6.7 (−5.4, 15.5)	7.3 (−5.7, 16.0)	3.2 (−7.2, 15.1)	0.623
Mean systolic blood pressure (mmHg, $\bar{\text{x}}$ ± s)	135.4 ± 18.4	139.0 ± 15.8	137.8 ± 17.2	0.365
Age (years, $\bar{\text{x}}$ ± s)	70 ± 12	71 ± 12	68 ± 13	0.360
Antiplatelet (*n*, %)	6 (6.1)	4 (6.2)	14 (6.3)	0.996
Statins (*n*, %)	7 (7.1)	8 (11.9)	21 (9.2)	0.564

*The OR of nocturnal blood pressure decline was presented as per 100%. Only Factors which were included in Table 4, were included in this table. Variables were presented as average ± standard deviation, median (interquartile range) or frequency (percentage), and statistically analyzed by ANOVA, Kruskal–Wallis H-test, Pearson chi test or Fisher's exact test*.

### Comparison of Nocturnal Blood Pressure Dipping Patterns Among Groups

**Figure 1:**
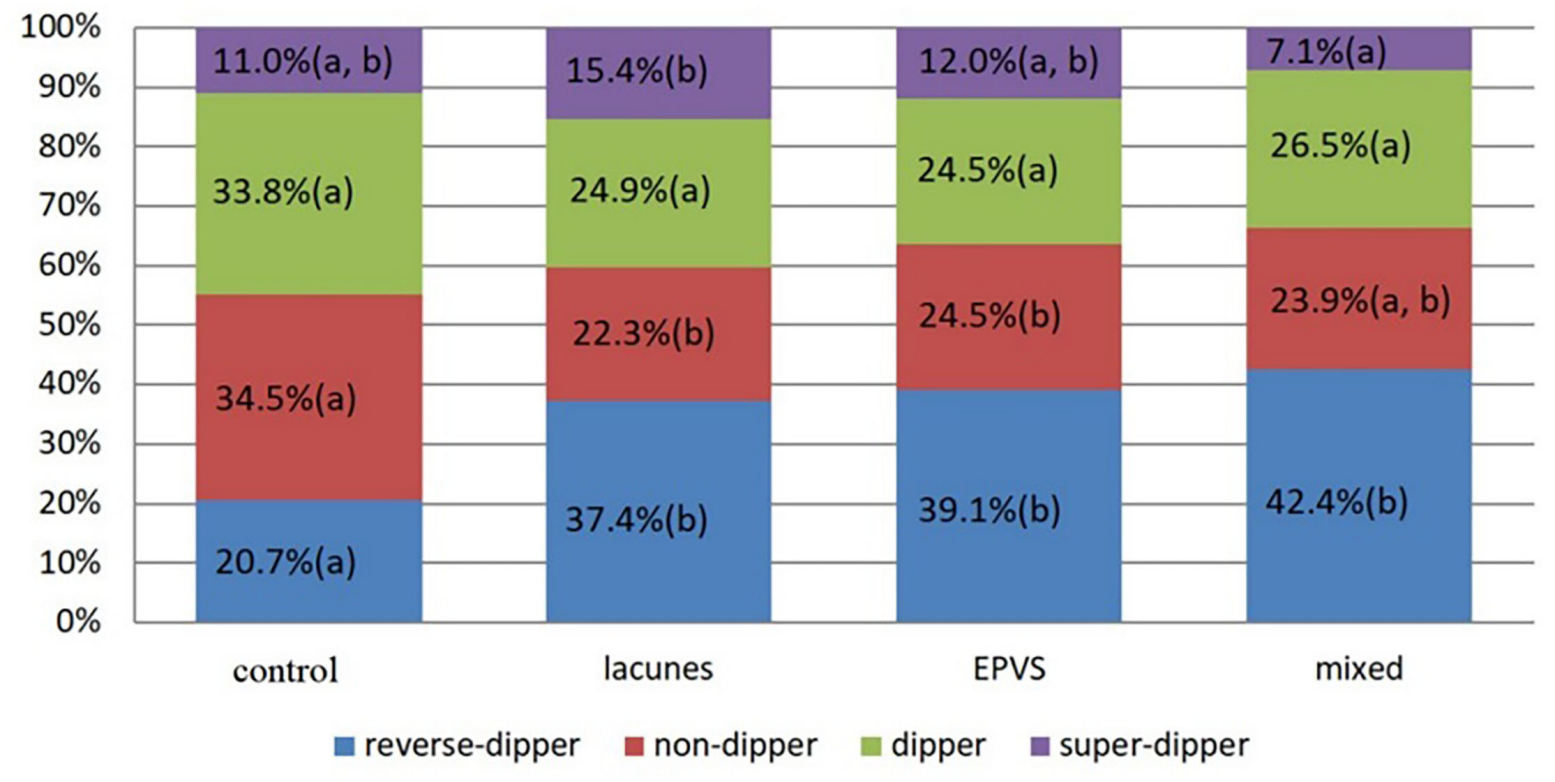
The length of the column represents the proportion of nocturnal blood pressure pattern in different groups. There was no significant difference (*P*-value vs. 0.05/6, by bonferroni correction) in proportion with a common letter (a or b) in each single color.

By the bar charts, it was shown that the frequency of reverse-dippers in the control group was significantly lower than that in other groups; the frequency of non-dippers in the lacunes group and EPVS group was significantly lower than that in the control group; the frequency of extreme-dippers in the EPVS group was significantly higher than that in the mixed group. Totally, the hypertensive patients with lacunes/EPVS are more likely to have abnormal nocturnal BP dipping patterns (Figure 1).

## Discussion

In this study, the associations of common risk factors and dipping patterns with lacunes and EPVS were analyzed in patients with hypertension. The findings were as follows. (1) Nocturnal BP decline showed an independent negative correlation with both lacunes and EPVS while mSBP level showed an independent positive correlation with them (*P* < 0.05). (2) The frequency of reverse-dippers in the control group was significantly lower than that in other groups; the frequency of non-dippers in the lacunes group and EPVS group was significantly lower than that in the control group; the frequency of extreme-dippers in the EPVS group was significantly higher than that in the mixed (lacunes with EPVS) group (*P* < 0.05).

Although lacunes and EPVS could exist simultaneously, they have different pathological mechanisms. Lacunes mostly originate from branch artery occlusion (15) and are distributed in the subcortical region, especially the basal ganglia (8). Previous studies reported hypertension, diabetes mellitus, hyperlipemia, and smoking are risk factors for lacunes. The increase of homocysteine was also found to be associated with lacunes in the basal ganglia region (16) and a higher imaging burden of CSVD (17). The findings of our research are consistent with previous researches. There is debate regarding the use of antiplatelets in patients with lacunes (18). Our study indicated a protective effect of antiplatelet on lacunes.

Perivascular spaces are important structures composed of the basal membrane of the peripheral vascular wall, pia mater, astrocytes, etc. The lumen is filled with interstitial fluid, participating in the substance exchange, especially the removal of the metabolic waste of the central nervous system (19). With the long-term effects of certain risk factors, EPVS will gradually form and become visible on MRI images. Age is one of the most significant risk factors for EPVS (20). Hypertension was also found to be associated with EPVS in basal ganglia (20), but not with EPVS in centrum semiovale (20). Regarding diabetes mellitus, some studies discovered that it might increase the score of EPVS (21), while others did not (22). In our study, we found that diabetes mellitus was not the independent risk factor for EPVS. In addition, a randomized, double-blind, and placebo-controlled trial revealed that statins therapy might be positively associated with delayed lacunes, EPVS, and the high signal in white matter but not with cerebral microbleeds (23). Our results were consistent with previous findings that statins were the negative correlation factor for both lacunes and EPVS (22). Additionally, it was also found that antiplatelet might have a protective role for EPVS in our study, which needs to be further verified. However, we find no significant difference between different types of EPVS groups, while some reported the discrepancies (7).

Long-term hypertension may cause damage to the vascular endothelium, arteriole hyalinosis, and blood-brain barrier leakage (24, 25), which is one of the most significant risk factors for CSVD. Abnormal dipping patterns are common in patients with hypertension. Several studies have reported the effects of dipping patterns on CSVD. One study found that compared to daytime BP, the nighttime BP was more strongly associated with the development of CSVD (WMHs, lacunes, CMBs) in patients with type 1 diabetes mellitus (26). Another follow-up study discovered that abnormal dipping patterns, especially the morning surge in BP, played an important role in CSVD (especially CMBs) (27). By means of meta-analysis, Anthipa found that compared with dippers, the reverse-dippers had a higher risk for WMHs and acute lacunar infarction and so did non-dippers for acute lacunar infarction (28). It is also reported that higher SBP levels were independently associated with EPVS in basal ganglia, but not in centrum semiovale (7). We demonstrated that factors including mSBP and dipping patterns might be significantly important for lacunes, EPVS, and mixed lesions. Additionally, the frequency of reverse-dippers was significantly higher in the lacunes group, EPVS group, and mixed group than that in the control group. The frequency of non-dippers was significantly lower in the lacunes group and EPVS group when compared to the control group, and the frequency of extreme-dippers in the EPVS group was significantly higher than in the mixed group. In total, the hypertensive patients with lacunes/EPVS are more likely to have abnormal nocturnal BP dipping patterns. These results suggested that for patients with hypertension, it might be essential to not only control the mSBP level, but also make a personalized schedule for antihypertensive drugs, or named chronotherapy (for example, let the patients take anti-hypertensive medications at night). The chronotherapy might have an important role in reducing the degree of lacunes and EPVS, and other cerebral small vessel diseases, and improving the prognosis for patients with cerebral small vessel diseases (4).

The limitations of this study were as follows: (1) sleep in a hospital might be more likely to be disturbed than at home and (2) the sleep-associated evaluation parameters were not studied. Most studies have shown that the clearance of many harmful substances (such as the amyloid-beta) in the perivascular spaces tended to occur at night, which was associated with both the quality and the duration of sleep (29). Additionally, obstructive sleep apnea was one of the common causes of abnormal dipping patterns (30).

## Conclusion

In hypertensive patients, mSBP levels and abnormal dipping patterns were associated with lacunes and EPVS.

## Data Availability Statement

The raw data supporting the conclusions of this article will be made available by the authors, without undue reservation.

## Ethics Statement

The studies involving human participants were reviewed and approved by the Medical Ethics Committee of Ruijin Hospital. Written informed consent to participate in this study was provided by the patients/participants or patients/participants' legal guardian/next of kin.

## Author Contributions

YG wrote the manuscript. WD analyzed the data and made the tables and figures. BZ and JHa read the images of MRI as senior radiologists. YF and FS was responsible for statistics and analysis. DY and JS was responsible for collecting the data. JHu checked and revised the manuscript. YF designed the study, guided the writing, and submitted the paper. All authors contributed to the article and approved the submitted version.

## Funding

This research was funded by the National Natural Science Foundation of China (No. 81901180) and the Natural Science Foundation of Shanghai (20ZR1434200).

## Conflict of Interest

The authors declare that the research was conducted in the absence of any commercial or financial relationships that could be construed as a potential conflict of interest.

## Publisher's Note

All claims expressed in this article are solely those of the authors and do not necessarily represent those of their affiliated organizations, or those of the publisher, the editors and the reviewers. Any product that may be evaluated in this article, or claim that may be made by its manufacturer, is not guaranteed or endorsed by the publisher.
